# Extended-Spectrum Beta-Lactamase Producing-*Escherichia coli* Isolated From Irrigation Waters and Produce in Ecuador

**DOI:** 10.3389/fmicb.2021.709418

**Published:** 2021-10-04

**Authors:** Lorena Montero, Jorge Irazabal, Paul Cardenas, Jay P. Graham, Gabriel Trueba

**Affiliations:** ^1^Instituto de Microbiología, Colegio de Ciencias Biológicas y Ambientales, Universidad San Francisco de Quito, Quito, Ecuador; ^2^Agrocalidad, Agencia de Regulación y Control Fito y Zoosanitario, Quito, Ecuador; ^3^Environmental Health Sciences Division, University of California, Berkeley, Berkeley, CA, United States

**Keywords:** fresh produce, irrigation water, ESBL *E. coli*, CTX-M, Extended-spectrum beta-lactamase (ESBL)

## Abstract

In cities across the globe, the majority of wastewater – that includes drug resistant and pathogenic bacteria among other contaminants – is released into streams untreated. This water is often subsequently used for irrigation of pastures and produce. This use of wastewater-contaminated streams allows antibiotic-resistant bacteria to potentially cycle back to humans through agricultural products. In this study, we investigated the prevalence of extended-spectrum β-lactamase (ESBL)-producing *Escherichia coli* isolated from produce and irrigation water across 17 provinces of Ecuador. A total of 117 vegetable samples, 119 fruit samples, and 38 irrigation water samples were analyzed. Results showed that 11% of the samples were positive for *E. coli* including 11 irrigation water samples (29%), and samples of 13 vegetables (11%), and 11 fruits (9%). Among the 165 *E. coli* isolates cultured, 96 (58%) had the ESBL phenotype, and 58% of ESBL producing *E. coli* came from irrigation water samples, 11% from vegetables, and 30% from fruits. The *bla*_CTX–M__–__55_, *bla*_CTX–M 65_, and *bla*_CTX–M 15_ genes were the most frequently found gene associated with the ESBL phenotype and coincided with the *bla*_CTX–M_ alleles associated with human infections in Ecuador. Three isolates had the mcr-1 gene which is responsible for colistin resistance. This report provides evidence of the potential role of irrigation water in the growing antimicrobial resistance crisis in Ecuador.

## Introduction

The rise of antimicrobial resistance (AMR) is one of the most serious biological threats facing modern society, and the inability to treat bacterial infections is already occurring in many nosocomial infections (Frieri et al., 2017). The World Health (WHO) has listed extended spectrum β-lactamase-producing Enterobacteriaceae (ESBL-E) as the most critical antimicrobial resistant microorganisms, among the “Highest Priority” pathogens due to the increasing prevalence in humans and livestock (Yassin et al., 2017; Shrivastava et al., 2018; Li et al., 2019; Murray et al., 2021).

Globally, the majority of wastewater produced by urban settlements goes into streams without prior treatment. Only 20% of produced wastewater receives proper treatment (UNESCO, 2012), and the capacity to treat wastewater often depends on the income level of the country; treatment capacity is 70% of the generated wastewater in high-income countries, compared to ∼8% in low-income countries (Sato et al., 2013). This phenomenon is rising as urban populations grow and developing countries increasingly install pipes to channel wastewater away from communities, even before the development of wastewater treatment plants. The wastewater comes from diverse sources (e.g., homes, hospitals, and animal processing plants, etc.) and contains large quantities of antibiotic resistant bacteria (ARB), often carrying antimicrobial resistance to last-line antimicrobials, such as carbapenems (Lin et al., 2020).

These antimicrobial resistant bacteria (ARB) can cycle back to humans when wastewater-contaminated streams are used to irrigate produce or provide water to food animals (FAO and WHO, 2008; Leff and Fierer, 2013; Pigłowski, 2019); one recent example is the finding of New Delhi metallo-β-lactamases–type carbapenem-resistant *Escherichia coli* in water, domestic food animals, and humans (carbapenem, a last-line drug, is used exclusively in human medicine) (Li et al., 2019; Murray et al., 2021). Many antibiotic-resistant Enterobacterales, members of the intestinal microbiome (including *E. coli*), can survive and multiply in the environment (Vasco et al., 2015; Guerrero et al., 2020) and may colonize humans and domestic animals through the fecal-oral route of transmission. Plasmids and other mobile genetic elements (MGEs) carrying AMR genes promote the dissemination of AMR among intestinal bacteria in the intestine of vertebrates (Bonardi and Pitino, 2019), and this cycle is fundamentally captured in the One Health concept. Produce contamination can happen before pre-harvest (i.e., through contaminated irrigation water or manure fertilization) (Beuchat, 1996; Iwu and Okoh, 2019), as well as post-harvest (i.e., by washing, handling and processing food) with irrigation water (Murray et al., 2017).

Wastewater-impacted irrigation water has been identified as the main source of contamination for fresh produce with pathogenic microorganisms and ARB (Njage and Buys, 2015; Gekenidis et al., 2018a). The fecally contaminated produce can transfer ARB to the consumer especially when the produce is consumed fresh and uncooked (Pesavento et al., 2014; Araújo et al., 2017; Hölzel et al., 2018). Besides contributing to the spread of pathogens, irrigation water may potentially play a leading role in the dissemination of ARB (Moore et al., 2010; Hong et al., 2013; Gekenidis et al., 2018b; Vital et al., 2018).

The production of extended-spectrum β-lactamases (ESBL) is one of the most important mechanisms of antibiotic resistance in Enterobacteriaceae. ESBL genes can be divided into 4 groups: TEM, SHV, OXA, and CTX-M types (Bush and Jacoby, 2010); CTX-M type is the most prevalent of ESBLs described (Rossolini et al., 2008; Bevan et al., 2017). Enterobacteriaceae members are the most common bacterial agents causing foodborne outbreaks associated with the consumption of fresh produce (Cooper et al., 2007; Kilonzo-Nthenge et al., 2018; Al-Kharousi et al., 2019; McDaniel and Jadeja, 2019; Motlagh and Yang, 2019). Pathogenic *E. coli* is a key bacterium in foodborne illnesses, and commensal *E. coli* is a common indicator organism of fecal contamination in aquatic systems (Edberg et al., 2000; Rochelle-Newall et al., 2015; Motlagh and Yang, 2019). *E. coli* is also recognized as an important species in the spread of ARB, mainly due to a high aptitude to acquire genetic information through horizontal gene transfer (Grasselli et al., 2008; Hasegawa et al., 2018; Marlène et al., 2020).

In Ecuador, an upper middle-income country, wastewater is almost entirely released untreated into streams; these streams often serve as irrigation water for produce and food-animal agriculture (Ortega-Paredes et al., 2020a, b). There are few studies about the dissemination of ESBL-*E. coli* from irrigation water to produce (Ben Said et al., 2015; Vital et al., 2018); most of the studies have been carried out in fresh produce from retail centers and groceries (Bhutani et al., 2015; Faour-Klingbeil et al., 2016; Ortega-Paredes et al., 2018; Al-Kharousi et al., 2019; Yang et al., 2019; Colosi et al., 2020; Richter et al., 2020; Song et al., 2020). The aim of this study was to build upon the previous literature to understand the relationship between ARB in irrigation water and ARB on fresh produce obtaining samples from farms and their irrigation water. The study focused on the occurrence of extended spectrum β-lactamase producing *E. coli* in 17 provinces of Ecuador.

## Materials and Methods

### Study Areas

**FIGURE 1:**
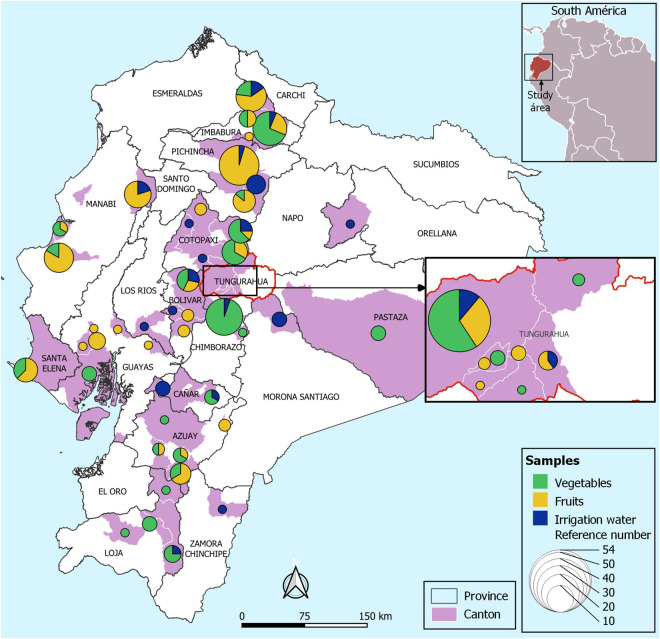
Map of Ecuador showing the sampling locations. Map of sampling locations of irrigation water, fruits and fresh produce. The circles represent the total number of samples according to the color assigned to each sample (vegetables: green, fruits: yellow, and irrigation water: blue) collected in each canton (pink).

This study was carried out in the following provinces of Ecuador: Manabí, Bolívar, Cañar, Loja, Guayas, Pastaza, Tungurahua, Pichincha, Azuay, Chimborazo, Cotopaxi, Imbabura, Santa Elena, Los Ríos, Morona Santiago, Orellana, and Zamora Chinchipe provinces which are mainly agrarian (Figure 1). The samples correspond to those that are collected as part of the national surveillance program that aims to monitor microbiological indicators and pathogens in the food supply (“Programa Nacional de Vigilancia de Microorganismos de Higiene y Control de Microorganismos Patógenos, para la Vigilancia Epidemiológica de Enfermedades Transmitidas por Alimentos de Origen Agrícola y Pecuario del país – PNVCH”).

### Sampling Fresh Produce

Fresh fruits and vegetables (representing 20 types) were obtained from agricultural farms in 17 provinces of Ecuador, from June to December 2019 (Figure 1). In total, 274 samples were analyzed (117 vegetables, 119 fruits were collected from agricultural farms. Among the vegetables consist of lettuce (*Lactuca sativa*, *n* = 43), onion (*Allium cepa*, *n* = 31), garlic (*Allium sativum*, *n* = 21), coriander (*Coriandrum sativum*, *n* = 17), cabbage (*Brassica oleracea* var. viridis, *n* = 2), spinach (*Spinacea oleracea*, *n* = 1), pepper (*Piper nigrum*, *n* = 1), tomato (*Solanum lycopersicum*, *n* = 1). The fruit samples correspond to cocoa (*Theobroma cacao*, *n* = 1), peach (*Prunus persica*, *n* = 2), strawberry (*Fregaria vesca*, *n* = 31), melon (*Cucumis melo* var. cantalupensis, *n* = 7), apple (*Malus domestica*, *n* = 1), banana (*Musa paradisiaca*, *n* = 13), blackberry (*Rubus ulmifolius*, *n* = 31), watermelon (*Citrullus lanatus*, *n* = 12), grape (*Vitis vinifera*, *n* = 1), and golden berry (*Physalis peruviana*, *n* = 20).

### Isolation of *Escherichia coli* From Irrigation Water and Produce

The farmers of each crop indicated the irrigation water they used, and this water (*n* = 37) was collected in sterile bottles and transported to the laboratory at approximately 8°C and processed within 10 h. Five hundred milliliters of water were filtered using a 0.45 μm pore membrane filter (Millipore, United States). The filter was then incubated in Chromocult^®^ coliform agar (Merck, Germany) overnight at 37°C, the apparent *E. coli* colonies were taken and seeded on MacConkey agar (Difco, United States) supplemented with ceftriaxone (2 mg/L) to identify the lactose positive colonies (a maximum of five colonies were picked from each plate) (Richter et al., 2020), colonies of presumptive *E. coli* were then tested for β-glucuronidase activity using Chromocult^®^ medium (Merck, Germany). All *E. coli* confirmed isolates from each sample were kept frozen at −80°C in Tryptic Soy Broth medium (Difco, United States) with 15% glycerol.

The vegetable samples were collected aseptically and refrigerated until analysis (within 12 h). Ten grams of the fresh produce were weighed and placed in a sterile plastic bag and incubated with 90 ml of peptone water (Faour-Klingbeil et al., 2016) for 30 min at room temperature. In the case of fruits such as watermelon and melon, the surface was swabbed, and the swab was placed in peptone water (described above). The next day 100 μl of the liquid was taken and cultured on MacConkey agar (Difco, United States) supplemented with ceftriaxone (2 mg/L) (Botelho et al., 2015). A maximum of five lactose positive colonies were selected from each plate sample and placed on Chromocult coliform agar after 24 h of incubation at 37°C, colonies of presumptive *E. coli*, positive for β-glucuronidase, were selected for additional analyses (Lange et al., 2013). All isolates confirmed to be *E. coli* from each sample were kept frozen at −80°C in Tryptic Soy Broth medium (Difco, United States) with 15% glycerol.

### Antimicrobial Susceptibility Testing

Susceptibility tests were performed using the Kirby-Bauer method on Mueller-Hinton agar (Difco, United States) in accordance with Clinical and Laboratory Standards Institute (CLSI, 2019). Eleven antibiotics were used for testing and included: Cefazolin, CZ (30 μg); Ampicillin, AM (10 μg), Gentamicin, GM (10 μg), Imipenem, IPM (10 μg); Trimethropin-sulfamethoxazole, SXT (1.25/23.75 μg); Ceftazidime, CAZ (30 μg); Cefepime, FEP (30 μg); Ciprofloxacin, CIP (5 μg); Amoxicillin/Clavulanic acid, AmC (20/10μg); cefotaxime, CTX (30μg); and Tetracycline TE (30 μg). After 18 h of incubation, the *E. coli* strains were classified as susceptible, intermediate, or resistant according to the clinical interpretation criteria recommended by CLSI. *E. coli* ATCC 25922 was used as a quality control. To determine the ESBL phenotype, we carried out a diffusion disk method on Mueller Hinton agar as before using antibiotic susceptibility discs (Oxoid, United States) of CTX (30 μg), CAZ (30 μg). Our criterion to determine ESBL was CTX ≤ 27 mm; CAZ ≤ 22 mm (CLSI, 2019). Specifically, ESBL production was confirmed by growth in a medium with discs of ceftazidime (30 mcg) and ceftazidime + clavulanic acid (30 mcg + 10 mcg). An increase of ≥5 mm in zone of inhibition for ceftazidime + clavulanic acid compared to ceftazidime was confirmed as ESBL producers (CLSI, 2019).

### PCR Amplification for Detection of β-Lactamase Genes

When samples were positive for ESBL-producing *E. coli*, one to five isolates selected per sample for further analysis. A total of 96 isolates were tested for the following resistance genes: *bla*_SHV_, *bla*_TEM_, *bla*_CTX–M_, and *bla*_OXA_ (Table 1). Bacterial DNA was extracted by boiling (Dashti et al., 2009), and PCR amplification reactions were performed in a volume of 25 μl containing 12.5 μl of 2 × Qiagen Multiplex PCR Master Mix (Qiagen GmbH, Hilden, Germany), 0.2 μM concentrations of each primer, and 2 μl of DNA template. The cycling parameters were as follows: an initial denaturation at 95°C for 15 min; followed by 30 cycles of 94°C for 30 s, 62°C for 90 s, and 72°C for 60 s; and with a final extension at 72°C for 10 min. Amplification products were observed in agarose gel electrophoresis 1.5%, stained with Ethidium bromide at 100V for 45–60 min. The size of the amplified products was compared with the commercial (Invitrogen, United States) 100-bp ladder. The band size (bp) for each gene was: *bla*_SHV_, 237; *bla*_TEM_, 445; *bla*_CTX–M_, 593; and *bla*_OXA_: 813 (Fang et al., 2008).

**TABLE 1:** Primers used for detection of different β-lactamase genes in the multiplex PCR.

**Genes**	**Primer sequence (5′ to 3′)**	**Size (bp)**	**References**
*bla*_SHV_	CTT TAT CGG CCC TCA CTCAA AGG TGC TCA TCA TGG GAA AG	237	Fang et al., 2008
*bla*_TEM_	CGC CGC ATA CAC TAT TCT CAG AAT GA ACG CTC ACC GGC TCC AGA TTT AT	445	Monstein et al., 2007
*bla*_CTX–M_	ATG TGC AGY ACC AGT AAR GTK ATG GC TGG GTR AAR TAR GTS ACC AGA AYC AGC GG	593	Boyd et al., 2004
*bla*_OXA_	ACA CAA TAC ATA TCA ACTTCGC AGT GTG TTT AGA ATG GTG ATC	813	Ouellette et al., 1987

### DNA Sequencing and Analysis

Genomic DNA was extracted from the isolates using the Wizard^®^ Genomic DNA Purification (Promega, United States) according to the manufacturer’s instructions. Sequencing was carried out at the University of Minnesota Mid-Central Research and Outreach Center (Willmar, Minnesota) using a single 2 × 250-bp dual-index run on an Illumina MiSeq with Nextera XT libraries to generate ∼30- to 50-fold coverage per genome. Genome assembly of MiSeq reads for each sample was performed using SPAdes assembler with the careful assembly option and automated k-mer detection (Bankevich et al., 2012). The identification of genus and species of the isolates was carried out using fastANI (Jain et al., 2018) with a percentage >80% of identification. Acquired AMR genes, plasmid types were identified using ABRicate tool (version 0.8.13), Resfinder was the database used for the identification of resistance genes (Zankari et al., 2012); PlasmidFinder database for plasmid replicon identification (Carattoli et al., 2014).

### Phylogenetic Analysis

Pan-genomic analysis was carried out with Roary (Page et al., 2015); the core genome of the isolates analyzed was defined with at least 99%. A maximum likelihood phylogenetic tree with (1,000 bootstrap replicates) was created based on the core genomes of the isolates using RaxML-NG (Kozlov et al., 2019). The phylogenetic tree was visualized using iTOL (Letunic and Bork, 2019). Additionally, multilocus sequence typing (MLST) (Larsen et al., 2012), based on seven housekeeping genes (adk, fumC, gyrB, icd, mdh, purA, and recA) and core genome (cgMLST) (Hansen et al., 2021) were performed using the Center for Genomic Epidemiology website^1^. The isolates also were characterized by Clermont phylogenetic typing by EzClermont web (Waters et al., 2020).

### Sequence Accession Number

The sequences were uploaded to Bioproject- NCBI under the following accession numbers: SAMN20872921, SAMN20872922, SAMN20872998, SAMN20873936, SAMN20873938, SAMN20873941, SAMN20873969, SAMN20873994, SAMN20874637, SAMN20875987, SAMN20875988, SAMN20875992, SAMN20875994, SAMN20875998, SAMN20879008, SAMN20879962, SAMN20879963, SAMN20879975, SAMN20879976, SAMN20880112, SAMN20880135, SAMN20880136, SAMN20881008, SAMN20881023, SAMN20881078, SAMN20881101, SAMN20881102, SAMN20881103, SAMN20881104, SAMN20881105, SAMN20881397, SAMN20881398, SAMN20881399, SAMN20881400, SAMN20882115, SAMN20882121, SAMN20882132, SAMN20882145, SAMN20882146, SAMN20882147, SAMN20882148, SAMN20882149, SAMN20883143, SAMN20883144, SAMN20883145, SAMN20883146, SAMN20883147, SAMN20884528, SAMN20884547, SAMN20884549, SAMN20886717, SAMN20887874, SAMN20887881, SAMN20887882, SAMN20887901, SAMN20887904, SAMN20887915, SAMN20887924, SAMN20887927, SAMN20887932, SAMN20887933, SAMN20888904, SAMN20888908, SAMN20888911, SAMN20888912, SAMN20888913, SAMN20888914, SAMN20888915, SAMN20888916, SAMN20888921, SAMN20888932, SAMN20888933, SAMN20888934, SAMN20888941, SAMN20888958, SAMN20888959, SAMN20888960, SAMN20888962, SAMN20890819, SAMN20891007.

## Results

### Prevalence of *Escherichia coli*

In total, 274 samples were collected, including 117 vegetable samples, 119 fruit samples, and 38 irrigation water samples. Across all samples, a total of 30 (11%) were positive for *E. coli;* 11 of the irrigation water samples had *E. coli* (29%, 11/38), 13 vegetables samples had *E. coli* (11%, *n* = 13), and 11 fruits (9%, *n* = 11). In total, 165 isolates of *E. coli* were recovered from 30 samples.

### Antimicrobial Susceptibility Testing

Ninety-six isolates (58% *n* = 96) showed extended-spectrum beta-lactamases (ESBL) phenotype according to the CLSI protocols; 58% of *E. coli* isolates from irrigation water were ESBL-producers, 11% from vegetables, and 30% from fruits. ESBL-*E. coli* were isolated from garlic (2 isolates), onion (9 isolates), strawberry (10 isolates), blackberry (4 isolates), banana (14 isolates), and golden berry (1 isolate).

The rate of resistance was high; more than 80% of recovered *E. coli* isolates were resistant to cefazolin, ampicillin, and cefotaxime. In the case of the *E. coli* isolates from irrigation water, 100% of the isolates were resistant to ampicillin and cefazolin. In addition, these isolates had a high prevalence of resistance to cefotaxime (96%), tetracycline (79%), and cefepime (84%) (Table 2).

**TABLE 2:** Antibiotic susceptibility profiles of isolates ESBL- *E.coli* from irrigation water, vegetables, and fruits.

**Antimicrobial categories**	**Antibiotics**	**Irrigation water *n* = 56 (frequency/percent)**			**Vegetables *n* = 11 (frequency/percent)**			**Fruits *n* = 29 (frequency/percent)**		
		***R***	***S***	**I/SDD**	***R***	***S***	**I/SDD**	***R***	***S***	**I/SDD**
Cephalosporins	Cefazolin	56/100	0/0	0/0	11/100	0/0	0/0	29/100	0/0	0/0
Penicillins	Ampicillin	56/100	0/0	0/0	11/100	0/0	0/0	29/100	0/0	0/0
Aminoglycosides	Gentamicin	17/30	39/70	0/0	7/64	4/36	0/0	15/52	13/45	1/3
Carbapenems	Imipenem	2/4	49/88	5/9	0/0	10/91	1/9	0/0	20/69	9/31
Sulfonamides	Trimethropin/Sulfamethoxazole	36/64	18/32	2/4	10/91	1/9	0/0	21/72	8/28	0/0
Cephalosporins	Ceftazidime	25/45	10/18	21/38	7/64	0/0	4/36	19/66	0/0	10/34
Cephalosporins	Cefepime	47/84	2/4	7/13	10/91	0/0	1/9	22/76	0/0	7/24
Fluoroquinolones	Ciprofloxacin	36/64	10/18	10/18	7/64	2/18	2/18	15/52	9/31	5/17
Aminopenicillin + inhibitor of betalactamase	Amoxicillin/clavulanic acid	17/30	23/41	16/29	6/55	1/9	4/36	22/76	5/17	2/7
Cephalosporins	Cefotaxime	54/96	1/2	1/2	11/100	0/0	0/0	29/100	0/0	0/0
Tetracyclines	Tetracycline	44/79	12/21	0/0	11/100	0/0	0/0	29/100	0/0	0/0

*R, resistant; I, intermediate; S, susceptible; SDD, susceptible-dose dependent in the case of cefepime; *n*, number of isolates tested.*

One hundred percent of the *E. coli* isolates from vegetables and fruits were resistant to ampicillin and cefazolin, cefotaxime, and tetracycline. Ninety-one percent of *E.coli* isolates from vegetables were resistant to cefepime. Two ESBL isolates from irrigation water presented resistance to the critically important class carbapenems, however no carbapenemase gene was detected. Additionally, we observed 33 resistance profiles across all of the extended spectrum beta-lactamase-producing *E. coli* isolates. The resistance profiles with the highest number of isolates are summarized in Table 3. In addition, 94% (90 of 96) of the *E. coli* ESBL isolates presented multi-drug resistant (MDR) patterns, with non-susceptible to at least one antibiotic in three or more antimicrobial categories (Magiorakos et al., 2012).

**TABLE 3:** The sixteen most common resistance profiles for ESBL-*E. coli* isolated from water, vegetables, and fruits in Ecuador.

**Resistance profiles**	**Produce/Fruits**	**Irrigation water**	**Total**
CZ-AM-GM-SXT-CAZ-FEP-CIP-AmC-CTX-TE	14	4	18
CZ-AM-FEP-CTX-TE	1	5	6
CZ-AM-SXT-CAZ-FEP-CIP-CTX-TE	0	4	4
CZ-AM-SXT-CAZ-FEP-CIP-AmC-CTX-TE	2	1	3
CZ-AM-GM-SXT-CAZ-FEP-CIP-CTX-TE	0	4	4
CZ-AM-GM-SXT-FEP-CIP-CTX-TE	1	4	5
CZ-AM-SXT-FEP-CIP-CTX-TE	0	4	4
CZ-AM-SXT-FEP-CIP-AmC-CTX-TE	4	2	6
CZ-AM-SXT-CAZ-FEP-CTX-TE	3	0	3
CZ-AM-CAZ-FEP-CTX-TE	1	2	3
CZ-AM-GM-CAZ-CTX-TE	2	0	2
CZ-AM-SXT-FEP-AmC-CTX-TE	2	1	3
CZ-AM-SXT-FEP-CTX-TE	4	0	4
CZ-AM-GM-CAZ-AmC-CTX-TE	4	0	4
CZ-AM-SXT-FEP-AmC-CTX	0	2	2
CZ-AM-SXT-CIP-CTX-TE	0	2	2

*CZ, cefazolin; AM, ampicillin; GM, gentamicin; IPM, imipenem; SXT, trimethropin-sulfamethoxazole; CAZ, ceftazidime; FEP, cefepime; CIP, ciprofloxacin; AmC, amoxicillin/Clavulanic acid; CTX, cefotaxime; TE, tetracycline.*

### Genotypes of Extended-Spectrum β-Lactamase – *Escherichia coli*

We obtained high-quality genome sequences of 80 ESBL-*E. coli* isolates. MLST analysis using 7 housekeeping genes showed that 80 isolates were assigned to 37 known STs, whereas 7 isolates represented 7 novel STs. ST10 was shared by 14% (*n* = 11) of isolates from three sources, with a different province of origin: irrigation water (Pichincha), onion (Tungurahua), banana (Manabí), and strawberry (Tungurahua). ST453 (5%, *n* = 4) and ST224 (8%, *n* = 6) were shared in two sources and in different provinces of origin of the sample: ST453 (banana = Manabí, irrigation water = Pichincha), ST224 (irrigation water = Pichincha and Zamora Chinchipe, banana = Manabí) (Table 4).

**TABLE 4:** Source and genetic characteristics of ESBL- *E. coli* isolates from different sources in Ecuador.

					**Relevant antimicrobial resistance genes**				
Sample (*)	**Source**	**Location**	**ST**	**cgST**	**CTX-M**	**TEM**	**SHV**	**OXA**	**mcr-1**
H505	Irrigation	Cañ-La Troncal	937	87149	55	141			
H719	Irrigation	Chim-Riobamba	617	93239	3				
H719	Irrigation	Chim-Riobamba	new7	143498	15	1	187	1	
H726	Irrigation	Imb-Ibarra	155	17156	55	141			
V662	Banana	Man-Portoviejo	10	15007	55	1			
V661.1	Banana	Man-Portoviejo	847	28793	55				
V662	Banana	Man-Portoviejo	6598	39050	8, 55	1			
V662	Banana	Man-Portoviejo	453	86226	8, 55	1			
V662	Banana	Man-Portoviejo	453	86226	55	1			
V662	Banana	Man-Portoviejo	453	86226	55	1	12		
V663 (3)	Banana	Man-Portoviejo	224	135673	55	1			1
V661.3	Banana	Man-Portoviejo	new3	136455	55	1	12		
HY1.3.3	Irrigation	Pich-Yaruquí	6027	2725	55	1			
HY6.5.3	Irrigation	Pich-Yaruquí	522	4492	55	1			
HP1.2	Irrigation	Pich-Yaruquí	10	5994	55,65	141			
HP6.4	Irrigation	Pich-Yaruquí	100	6271	15				
HY8.5.3	Irrigation	Pich-Yaruquí	131	9613			12		
HY3.4.3	Irrigation	Pich-Yaruquí	38	13889	9	1			
HY7.5.3	Irrigation	Pich-Yaruquí	206	17904	65	1			
HP1.4	Irrigation	Pich-Yaruquí	752	21656	65				
HY4.2.2	Irrigation	Pich-Yaruquí	224	29102	55	1			
V727 (2)	Strawberry	Pich-Yaruquí	new4	33815	65		12		
HP6.2	Irrigation	Pich-Yaruquí	1725	34210	55		5		
HY3.5	Irrigation	Pich-Yaruquí	1706	38416	15	1			
HP1.1	Irrigation	Pich-Yaruquí	155	40558	65				
HP4.3	Irrigation	Pich-Yaruquí	7290	43104	8				
HP7.2	Irrigation	Pich-Yaruquí	10	46675	55		12		
HP7.4	Irrigation	Pich-Yaruquí	10	46675	55	141	12		
HY2.4.2	Irrigation	Pich-Yaruquí	new2	79725	15				
HY4.4.2	Irrigation	Pich-Yaruquí	3944	80110	55	1			
HP4.4	Irrigation	Pich-Yaruquí	117	81681	55	141			
HP2.4	Irrigation	Pich-Yaruquí	117	82990	55	141			
HP6.3	Irrigation	Pich-Yaruquí	453	86226	55	141			
HY6 (2)	Irrigation	Pich-Yaruquí	540	96158	15	1			
HY1 (2)	Irrigation	Pich-Yaruquí	540	96158	15	1			
HP7	Irrigation	Pich-Yaruquí	124	96630	65				
HY6	Irrigation	Pich-Yaruquí	9580	96650	55	1			
HY2.3.3	Irrigation	Pich-Yaruquí	10	101136	15			1	
HY8.2.2	Irrigation	Pich-Yaruquí	9962	116134		1	12		
HP6.1	Irrigation	Pich-Yaruquí	1725	117316	55				
HY4.4 (2)	Irrigation	Pich-Yaruquí	205	117479	15	1			
HP6.5	Irrigation	Pich-Yaruquí	10340	117591	3	141			
HP2	Irrigation	Pich-Yaruquí	57	117853	55	141			
HP1.5	Irrigation	Pich-Yaruquí	57	117853	55	141			
V727.4	Strawberry	Pich-Yaruquí	new6	119048	65	176	12		
V727.5	Strawberry	Pich-Yaruquí	4541	119048	65		12		
HY6.5	Irrigation	Pich-Yaruquí	10	134002	55	1			
HY1.3.2	Irrigation	Pich-Yaruquí	2973	135505	55, 65	1			
HP1.3	Irrigation	Pich-Yaruquí	354	137556	55	1			
HY4.3.2	Irrigation	Pich-Yaruquí	224	138183	55	1			
HY1.1.4	Irrigation	Pich-Yaruquí	new1	138274		1			
HY5.2.1	Irrigation	Pich-Yaruquí	155	138689	55	1			
HY3.5.2	Irrigation	Pich-Yaruquí	155	138689	55	1			
HY1.4.3	Irrigation	Pich-Yaruquí	394	142214	15				
HY6.1.2	Irrigation	Pich-Yaruquí	69	144487	55	1			1
H579.2	Irrigation	Tun-Ambato	206	4018	65				
V696 (4)	Blackberry	Tun-Ambato	5044	32678	55	1			
V698 (3)	Strawberry	Tun-Ambato	10	38518	55	1			
V1140 (2)	Onion	Tun-Ambato	4204	55533	55, 65	1			
V1140	Onion	Tun-Ambato	4204	55533	55	1			
V427.5	Onion	Tun-Ambato	58	60063	55	1			
V469.5	Onion	Tun-Ambato	10	69259	55	1			
V1147 (2)	Garlic	Tun-Ambato	973	118630	3	1			
H579.1	Irrigation	Tun-Ambato	155	138689	55	1			
V427.2	Onion	Tun-Ambato	4368	142214	15				
H430	Irrigation	Zam-Yantzaza	224	135673	55	1			1

*Number of isolates with the same cgST obtained from the same sample. Tun, Tungurahua; Pich, Pichincha; Man, Manabi; Zam, Zamora; Imb, Imbabura; Cañ, Cañar; Chim, Chimborazo.

**FIGURE 2:**
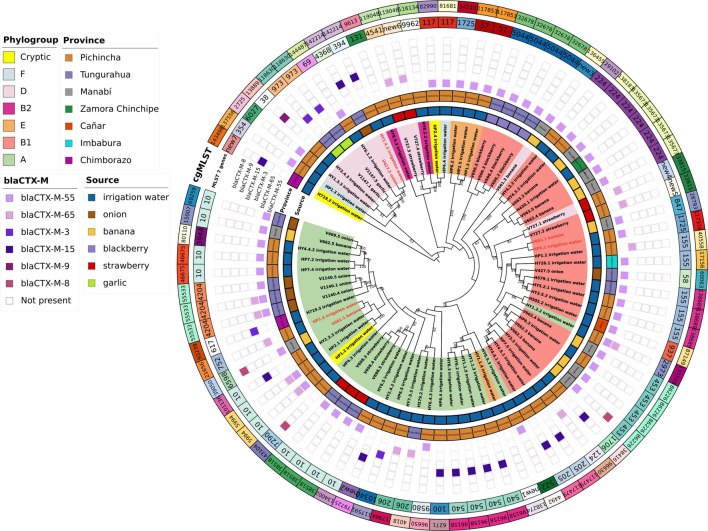
Frequency of allelic variants of AMR genes in *E. coli* from different sources.

The application of a cgMLST scheme showed 55 cgSTs, from which only 2, cgST86226 (banana, Manabí, *n* = 5; irrigation water Pichincha, *n* = 1) and cgST135673 (banana Manabí, *n* = 3; irrigation water, Zamora Chinchipe *n* = 1) were isolates from two different sources. Several isolates belonging to the same ST (based on 7 genes) were assigned to different cgSTs based on cgMLST and some of the isolates from the same sample had the same cgST. Additionally, we constructed a maximum likelihood tree based on the core genomes to compare the phylogeny of isolates of *E. coli* from the irrigation water, vegetables, and fruits (Figure 2). The phylogenetic analysis showed that all isolates with the same cgMLST and obtained from different sources differed in thousands of SNPs indicating that although the isolates were genetically close, they have been evolving apart for many years (Table 4 and Figure 2). The genomes of ESBL-*E. coli* isolates from irrigation and fresh produce did not cluster apart; instead the isolates form different sources seemed to share recent common ancestry (Figure 2).

When ESBL-*E.coli* isolates were characterized by Clermont phylogenetic typing, 38% (*n* = 30) isolates belonged to phylogroup A: irrigation water (*n* = 21), strawberry (*n* = 3), onion (*n* = 4), banana (*n* = 2). In phylogroup B1 accounted for 35% (*n* = 28) of isolates: irrigation water (*n* = 15), banana (*n* = 7), strawberry (*n* = 1), blackberry (*n* = 4), and onion (*n* = 1). In phylogroup D accounted for 14% of the isolates: irrigation water (*n* = 4), strawberry (*n* = 3), garlic (*n* = 2), onion (*n* = 1) and banana (*n* = 1). Phylogroups B2, E and F accounted for 3% (*n* = 2), 5% (*n* = 4) and 3% (*n* = 2) of isolates, respectively. Three (4%) isolates of irrigation water belonged to the cryptic lineage (Figure 2).

### Detection of β-Lactamase Genes

Ninety-six *E.*coli isolates phenotypically identified as ESBL, were tested by Multiplex PCR for genes encoding SHV, TEM, CTX-M, and OXA enzymes. The CTX-M gene was detected in 98% (94 of 96) of the isolates, followed by TEM 92% (88 of 96), SHV 28% (27 of 96), and OXA 1% (1/96). Additionally, combinations of genes were present: 64% had both CTX-M and TEM; and 26% had CTX-M, TEM, and SHV.

**FIGURE 3:**
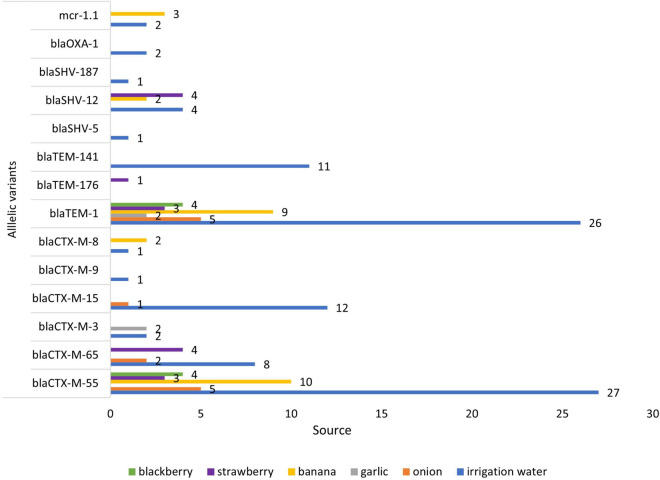
Phylogenetic tree of ESBL-*E.coli* sequences from irrigation water, fruits, and vegetables. Maximum likelihood phylogenetic tree of the core genomes of 80 ESBL-*E.coli* isolates from irrigation water, fruits, and vegetables. The labels show the isolate ID assigned according to the sample ID, the origin of the isolate is shown by source colors (irrigation water: blue, onion: brown, banana: yellow, blackberry: purple, strawberry: red, and garlic: green). The background colors highlighted on the branches of the tree indicate the seven identified phylogroups. Numbers represent bootstrap values using 1,000 pseudo-replicates.

The presence of AMR genes in the genome sequences of 80 *ESBL-E. coli* isolates was investigated by Resfinder. Several ESBL-encoding *bla*_CTX–M_ gene variants were distributed in isolates from irrigation water and fresh produce (Figure 3). Among the 80 *ESBL-E. coli* isolates, we identified allelic variants of *bla*_CTX–M_ in 77 (96%). The most common allelic variants were *bla*_CTX–M–__55_ in 49 isolates (64%) and the second most common allele was bla_CTX–M–__65_ in 14 isolates (18%) (Supplementary Table 1).

We found some discrepancies in some ESBL- *E.coli* isolates that were positive by PCR for some genes but negative by whole genome sequencing (WGS): 12 isolates for *bla*_TEM_ gene, 9 isolates for *bla*_SHV_ genes and *bla*_CTX–M_ in one gene. Additionally, 2 isolates showed *bla*_SHV_ and *bla*_TEM_ using WGS, but were negative by PCR. The WGS analysis of ESBL*-E. coli* allowed us to identify 2 isolates of *E. coli* from irrigation water and 3 isolates from banana with the presence of the *mcr-1* gene that confers resistance to colistin.

## Discussion

In this study, we found that irrigation water, fruit, and vegetables were contaminated with ESBL-*E. coli* and the highest percentage was found in irrigation water (58%), which confirms the important and emerging role that irrigation water, contaminated with wastewater, has in the spread of ARB and ESBL *E. coli* and ESBL genes. (Gekenidis et al., 2018a; Vital et al., 2018). The major ESBL gene was the _CTX–M_ (94 of 96 isolates) followed by *bla*_SHV_ 28% (27 of 96), and *bla*_OXA_ 1% (1of 96). The prevalence of *bla*_CTX–M_ type ESBL genes in irrigation water *E. coli* was 57%, followed by 15% in banana isolates. Additionally the most abundant allelic variants of *bla*_*CTX–M*_ found in vegetables, fruits and irrigation water (*bla*_CTX–M__55_, *bla*_CTX–M__65_, and *bla*_CTX–M__15_) (Table 4) are the same alleles found in children and domestic animals in Ecuador (Salinas et al., 2021), in rivers that cross cities (Ortega-Paredes et al., 2020a), and in bacteria from human infections in Ecuador (Cartelle Gestal et al., 2016; Soria Segarra et al., 2018). The presence of the same *bla*_CTX–M_ alleles in isolates from different sources provides strong evidence that these sources (irrigation water, domestic animals, and humans) are connected. The allelic variants of *bla*_CTX–M_ from isolates obtained from same European country, but from different (unconnected) sources, animal species or time periods, have been shown to be different (Day et al., 2019; Ludden et al., 2019).

Our genomic analysis showed that most strains obtained from irrigation water and produce were genetically different with 3 exceptions (HY1.4.3 and V427.2; HP6.1 and V661.1; HP1.4 and V662.1), however the number of SNPs between thes strains ranged from 9,332 to 20,310 suggesting that these strains have been evolving apart for many years (Table 4). As expected, some isolates from the same vegetable or fruit showed higher level of genetic closeness, for instance: V698.3 and V698.4 had 12 SNP; V663.4 and V663.5, 6 SNPs; V696.2 and V696.4, 13 SNPs; V1147.5 and V1147.1, 2 SNPs). Interstingly, 2 isolates obtaind from the same irrigation channel 1 month appart (HY3.5.2 and HY5.2.1) had 24 SNPs, suggesting that this strain was higly adapted to water. We did not find additional asociation of ESBL-*E.coli* clusters with provinces, which may indicate that different *E. coli* lineages have been widely distributed in the Ecuadorian territory (Figure 2).

These findings may indicated that *E. coli* populations in the environment are highly diverse (Day et al., 2019; Ludden et al., 2019) and *bla*_CTX–M_-genes are probably diseminating in the environmet mostly by mobile genetic elements and not so much by bacterial clones. The plasmids carrying *bla*_CTX–M_-genes disseminate efficiently by conjugation, even between bacteria belonging to different genera (Cantón et al., 2012). Transposable elements (such as IS*Ecp1*) are also very active in *bla*_CTX–M_-gene mobilization among different plasmids (Cantón et al., 2012). The activity of these MGEs conceals the source of origin of these antimicrobial resistance genes.

The majority of strains isolated from irrigation water and vegetables belonged to phylogroups A and B1 which are considered more generalists, found in most warm-blooded animals and environmental samples (Touchon et al., 2020). We found that some genetically close *E.coli* isolates, obtained from the same vegetable, had 1 or 2 additional antimicrobial resistance genes which may be a reflection of the dynamic process of antimicrobial resistance gene-turnover in the environment (Barrera et al., 2019).

The *bla*_CTX–M_ type of ESBL gene is of increasing concern globally (Bevan et al., 2017), and is the predominant ESBL gene in both community and hospital-acquired infections (Manyahi et al., 2017; Fils et al., 2021). A troubling feature of *bla*_CTX–M_-bearing plasmids is their ability to capture additional resistance determinants, including carbapenemase genes (Partridge et al., 2012; Potron et al., 2013). Further analysis is necessary to understand whether the plasmids carrying *bla*_CTX–M_ genes, in bacteria from irrigation water and produce, are the same as those circulating in bacterial isolates from human isolates.

In our study fruits, such as bananas, we hypothesize that their contamination was due to post-harvest processes in which the food is often washed in contaminated water and reused to wash several batches of the product. Although it is true, the skin of the product protects the fruit, the transmission of resistant bacteria can occur through contact and inadequate consumer hygiene (Harris et al., 2003; Hong et al., 2013; Kawamura et al., 2017; Murray et al., 2017; Hölzel et al., 2018).

We also found a higher prevalence of ARB in vegetables in farms than in retail markets in Ecuador (Ortega-Paredes et al., 2018). However, other reports from the Philippines, Lebanon, and Portugal have documented even higher levels (Faour-Klingbeil et al., 2016; Araújo et al., 2017; Vital et al., 2018). In most of the studies, the collection of produce samples has been carried out in groceries and wholesale markets, which makes it difficult to analyze sources of contamination (Bhutani et al., 2015; Yang et al., 2019; Colosi et al., 2020; Richter et al., 2020; Song et al., 2020). In this study, we collected produce and water from farms and their respective irrigation systems, which allowed us to study contamination at the source (i.e., not due to handling, transport, distribution, and processing). We found that MDR isolates were more prevalent in irrigation water isolates compared to fresh produce. Similar results were observed in the Philippines, where 58% of the *E. coli* isolates from irrigation water were MDR (Paraoan et al., 2017). The resistance to these antibiotics was also observed in *E. coli* isolates from irrigation water in other studies (Pignato et al., 2009; Ben Said et al., 2015; Vital et al., 2018).

Our study had some limitations; the number produce and fruit samples obtained in each location may not be representative of produce from other agricultural settings in Ecuador. Additionally, long-read sequencing of plasmids could not be carried out due to budgetary limitations.

We found evidence that fresh produce constitutes an important source of ESBL-*E. coli* and represents a route for the dissemination of resistance genes through the consumption of raw products (Rasheed et al., 2014; Hölzel et al., 2018; Al-Kharousi et al., 2019). We hypothesize that the main source of ABR contamination is irrigation water used for the cultivation of produce, which has been suggested by others as well (Pignato et al., 2009; Gekenidis et al., 2018b). In Ecuador, the lack of sewage treatment may lead to contamination of the food supply with ARB, mainly belonging to the Enterobacteriaceae family (Caicedo-Camposano et al., 2019; Ortega-Paredes et al., 2020a). Antibiotic resistant *E. coli* can transfer antibiotic resistance determinants not only to other strains of *E. coli*, but also to other species of potentially pathogenic bacteria within the gastrointestinal tract (Grasselli et al., 2008; Huddleston, 2014).

## Conclusion

We found a high prevalence of ESBL-*E. coli* on produce and in irrigation water; *bla*_CTX–M_ was the main ESBL gene in these isolates. Allelic variants of the *bla*_CTX–M_ gene found in irrigation channels and vegetables were the same as those observed in commensal *E. coli* from domestic animals, and commensal and pathogenic *E. coli* from humans, suggesting connection between these different sources. This paradigm poses the potential risk of further spreading ARB that are resistant to last-line antibiotics such as carbapenems, which are used exclusively in serious infections in hospitals (Sheu et al., 2019). In this case, resistance goes full circle, from humans to vegetables and fruits (potentially meat and dairy), and back to human populations (Murray et al., 2021). Greater investments are needed to support the development and installation of wastewater treatment systems throughout Ecuador, as well as in other low- and middle-income countries.

## Data Availability Statement

The raw data supporting the conclusions of this article will be made available by the authors, without undue reservation.

## Author Contributions

JI and LM: isolation of the *Escherichia coli* strains. LM: writing—original draft. JG, PC, and GT: review and editing. GT and LM: study design. All authors contributed to the article and approved the submitted version.

## Conflict of Interest

The authors declare that the research was conducted in the absence of any commercial or financial relationships that could be construed as a potential conflict of interest.

## Publisher’s Note

All claims expressed in this article are solely those of the authors and do not necessarily represent those of their affiliated organizations, or those of the publisher, the editors and the reviewers. Any product that may be evaluated in this article, or claim that may be made by its manufacturer, is not guaranteed or endorsed by the publisher.

## Footnotes

1
https://www.genomicepidemiology.org/
