# Corrigendum: Extended-Spectrum Beta-Lactamase Producing-*Escherichia coli* Isolated From Irrigation Waters and Produce in Ecuador

**DOI:** 10.3389/fmicb.2022.926514

**Published:** 2022-06-14

**Authors:** Lorena Montero, Jorge Irazabal, Paul Cardenas, Jay P. Graham, Gabriel Trueba

**Affiliations:** ^1^Instituto de Microbiología, Colegio de Ciencias Biológicas y Ambientales, Universidad San Francisco de Quito, Quito, Ecuador; ^2^Agrocalidad, Agencia de Regulación y Control Fito y Zoosanitario, Quito, Ecuador; ^3^Environmental Health Sciences Division, University of California, Berkeley, Berkeley, CA, United States

**Keywords:** fresh produce, irrigation water, ESBL *E. coli*, CTX-M, Extended-spectrum beta-lactamase (ESBL)

In the original article, there was a mistake in the legend for **Figure 3** as published. We included the word “host” by mistake. The correct legend appears below.

**Figure 3**. Phylogenetic tree of ESBL-*E.coli* sequences from irrigation water, fruits, and vegetables. Maximum likelihood phylogenetic tree of the core genomes of 80 ESBL-*E.coli* isolates from irrigation water, fruits, and vegetables. The labels show the isolate ID assigned according to the sample ID, the origin of the isolate is shown by source colors (irrigation water: blue, onion: brown, banana: yellow, blackberry: purple, strawberry: red, and garlic: green). The background colors highlighted on the branches of the tree indicate the seven identified phylogroups. Numbers represent bootstrap values using 1,000 pseudo-replicates.

In the original article, there was an error, the manuscript states that sequences were deposited in the European Nucleotide Archive but were deposited to Bioproject- NCBI.

A correction has been made to the Section **Materials and Methods**, “*Sequence Accession Number*,” paragraph one:

“The sequences were uploaded to Bioproject- NCBI under the following accession numbers: SAMN20872921, SAMN20872922, SAMN20872998, SAMN20873936, SAMN20873938, SAMN20873941, SAMN20873969, SAMN20873994, SAMN20874637, SAMN20875987, SAMN20875988, SAMN20875992, SAMN20875994, SAMN20875998, SAMN20879008, SAMN20879962, SAMN20879963, SAMN20879975, SAMN20879976, SAMN20880112, SAMN20880135, SAMN20880136, SAMN20881008, SAMN20881023, SAMN20881078, SAMN20881101, SAMN20881102, SAMN20881103, SAMN20881104, SAMN20881105, SAMN20881397, SAMN20881398, SAMN20881399, SAMN20881400, SAMN20882115, SAMN20882121, SAMN20882132, SAMN20882145, SAMN20882146, SAMN20882147, SAMN20882148, SAMN20882149, SAMN20883143, SAMN20883144, SAMN20883145, SAMN20883146, SAMN20883147, SAMN20884528, SAMN20884547, SAMN20884549, SAMN20886717, SAMN20887874, SAMN20887881, SAMN20887882, SAMN20887901, SAMN20887904, SAMN20887915, SAMN20887924, SAMN20887927, SAMN20887932, SAMN20887933, SAMN20888904, SAMN20888908, SAMN20888911, SAMN20888912, SAMN20888913, SAMN20888914, SAMN20888915, SAMN20888916, SAMN20888921, SAMN20888932, SAMN20888933, SAMN20888934, SAMN20888941, SAMN20888958, SAMN20888959, SAMN20888960, SAMN20888962, SAMN20890819, SAMN20891007.”

The original article contains texts identical to those found in another article published by our group.

A correction has been made to the Section **Materials and Methods**, “*DNA Sequencing and Analysis*:”

“Genomic DNA was extracted from the isolates using the Wizard^®^ Genomic DNA Purification (Promega, United States) according to the manufacturer's instructions. Sequencing was carried out at the University of Minnesota Mid-Central Research and Outreach Center (Willmar, Minnesota) using a single 2 × 250-bp dual-index run on an Illumina MiSeq with Nextera XT libraries to generate ~30- to 50-fold coverage per genome. Genome assembly of MiSeq reads for each sample was performed using SPAdes assembler with the careful assembly option and automated k-mer detection (Bankevich et al., 2012). The identification of genus and species of the isolates was carried out using fastANI (Jain et al., 2018) with a percentage >80% of identification. Acquired AMR genes, plasmid types were identified using ABRicate tool (version 0.8.13), Resfinder was the database used for the identification of resistance genes (Zankari et al., 2012); PlasmidFinder database for plasmid replicon identification (Carattoli et al., 2014).”

A correction has been made to the Section **Materials and Methods**, “*Phylogenetic Analysis*,” paragraph one:

“Pan-genomic analysis was carried out with Roary (Page et al., 2015); the core genome of the isolates analyzed was defined with at least 99%. A maximum likelihood phylogenetic tree with (1,000 bootstrap replicates) was created based on the core genomes of the isolates using RaxML-NG (Kozlov et al., 2019). The phylogenetic tree was visualized using iTOL (Letunic and Bork, 2019). Additionally, multilocus sequence typing (MLST) (Larsen et al., 2012), based on seven housekeeping genes (adk, fumC, gyrB, icd, mdh, purA, and recA) and core genome (cgMLST) (Hansen et al., 2021) were performed using the Center for Genomic Epidemiology website^1^. The isolates also were characterized by Clermont phylogenetic typing by EzClermont web (Waters et al., 2020).”

The authors apologize for these errors that do not change the scientific conclusions of the article in any way. The original article has been updated.^1^

## Publisher's Note

All claims expressed in this article are solely those of the authors and do not necessarily represent those of their affiliated organizations, or those of the publisher, the editors and the reviewers. Any product that may be evaluated in this article, or claim that may be made by its manufacturer, is not guaranteed or endorsed by the publisher.

## Footnotes

^1^ https://www.genomicepidemiology.org/
