# Supplementary material for: Extended-Spectrum Beta-Lactamase Producing-Escherichia coli Isolated From Irrigation Waters and Produce in Ecuador
Source: Front Microbiol. 2021 Oct 4;12:709418. doi: 10.3389/fmicb.2021.709418 (PMC8521160; doi:10.3389/fmicb.2021.709418)
Supplement: Supplementary file 1 [file Table_1.docx]

**Table S1.** Percentage of allelic variants of AMR genes depending on the isolation source

| **Source** | **Allelic variants (n”; %)** | | | | | | | | | | | | | |
| --- | --- | --- | --- | --- | --- | --- | --- | --- | --- | --- | --- | --- | --- | --- |
|  | **bla_CTX-M-55_** | **bla_CTX-M-65_** | **bla_CTX-M-3_** | **bla_CTX-M-15_** | **bla_CTX-M-9_** | **bla_CTX-M-8_** | **bla_TEM-1_** | **bla_TEM-176_** | **bla_TEM-141_** | **bla_SHV-5_** | **bla_SHV-12_** | **bla_SHV-187_** | **bla_OXA-1_** | **mcr-1.1** |
| **irrigation water** | 27”; 55 | 8”; 57 | 2”; 50 | 12”; 92 | 1”;1 00 | 1”; 33 | 26”; 53 | 0”; 0 | 11”; 100 | 1”; 100 | 4”; 40 | 1”; 100 | 2”; 100 | 2”; 40 |
| **onion** | 5”; 10 | 2”; 14 | 0”; 0 | 1”; 8 | 0”; 0 | 0”; 0 | 5”; 10 | 0”; 0 | 0”; 0 | 0”; 0 | 0”; 0 | 0”; 0 | 0”; 0 | 0”; 0 |
| **garlic** | 0”; 0 | 0”; 0 | 2”; 50 | 0”; 0 | 0”; 0 | 0”; 0 | 2”; 4 | 0”; 9 | 0”; 0 | 0”; 0 | 0”; 0 | 0”; 0 | 0”; 0 | 0”; 0 |
| **banana** | 10”; 20 | 0”; 0 | 0”; 0 | 0”; 0 | 0”; 0 | 2”; 67 | 9”; 18 | 0”; 0 | 0”; 0 | 0”; 0 | 2”; 20 | 0”; 0 | 0”; 0 | 3”; 60 |
| **strawberry** | 3”; 6 | 4”; 29 | 0”; 0 | 0”; 0 | 0”; 0 | 0”; 0 | 3”; 6 | 1”; 100 | 0”; 0 | 0”; 0 | 4”; 20 | 0”; 0 | 0”; 0 | 0”; 0 |
| **blackberry** | 4”; 8 | 0”; 0 | 0”; 0 | 0”; 0 | 0”; 0 | 0”; 0 | 4”; 8 | 0”; 0 | 0”;0 | 0”; 0 | 0”; 0 | 0”; 0 | 0”; 0 | 0”; 0 |

n”: number of isolates for each variant, % = percentage based on the total number of isolates for each variant
